# Family satisfaction in the ICU: enhancing patient experience

**DOI:** 10.1186/cc12482

**Published:** 2013-03-19

**Authors:** E Kursumovic, J Bilinska, A Molokhia

**Affiliations:** 1 University Hospital Lewisham, London, UK

## Introduction

Following recommendations made by the Scottish Intensive Care Society [1], we have introduced an annual Family Satisfaction Survey in the intensive care unit (FS-ICU), in anticipation that this will become an integral part of how we measure, improve and strive to advance patient care in the future. We aim to recognise factors impacting patient care and highlight areas for improvement. Improving understanding of patients' needs is currently a UK national goal for CQUIN 2011/2012. An estimated £6.9 billion per year can be saved by reaching these goals [2] and a positive patient experience results in improved long-term outcomes and shorter hospital stays [2].

## Methods

We performed a study to assess Family Satisfaction over a 10-week period in a 16-bed critical care unit (CCU). A modified version of an FS-ICU published in US and Canadian studies [3] was distributed to up to two family members per patient. Five-point Likert scale responses were linearly transformed to give percentage scores. Higher values represented a greater degree of satisfaction.

## Results

We received and analysed 32 completed surveys. Seventy-eight per cent of relatives reported that the treatment of the patient was excellent, including symptom control such as pain, breathlessness and agitation (72%, 69%, 100%, respectively). Ninety-one per cent felt the care and frequency of communication provided by nurses was excellent or very good, compared with 81% by doctors. Only 9% of family members were offered spiritual support during their CCU experience. Forty-seven per cent felt they would have liked more involvement in the decision-making process.

## Conclusion

The vast majority of relatives rated their overall experience on CCU as excellent or very good. The study highlighted two main areas for improvement: provision of spiritual support as well as family and patient involvement in the decision-making process. We will arrange multi-disciplinary teaching sessions focusing on the positive impact, and therefore importance of patient satisfaction. A poster providing information about available spiritual support will be displayed around our CCU. Further studies are required to evaluate these measures.
